# Electrocortical correlates of human level-ground, slope, and stair walking

**DOI:** 10.1371/journal.pone.0188500

**Published:** 2017-11-30

**Authors:** Trieu Phat Luu, Justin A. Brantley, Sho Nakagome, Fangshi Zhu, Jose L. Contreras-Vidal

**Affiliations:** Noninvasive Brain-Machine Interface System Laboratory, Dept. of Electrical and Computer Engineering, University of Houston, Houston, TX, United States of America; Wadsworth Center, UNITED STATES

## Abstract

This study investigated electrocortical dynamics of human walking across different unconstrained walking conditions (i.e., level ground (LW), ramp ascent (RA), and stair ascent (SA)). Non-invasive active-electrode scalp electroencephalography (EEG) signals were recorded and a systematic EEG processing method was implemented to reduce artifacts. Source localization combined with independent component analysis and k-means clustering revealed the involvement of four clusters in the brain during the walking tasks: Left and Right Occipital Lobe (LOL, ROL), Posterior Parietal Cortex (PPC), and Central Sensorimotor Cortex (SMC). Results showed that the changes of spectral power in the PPC and SMC clusters were associated with the level of motor task demands. Specifically, we observed α and β suppression at the beginning of the gait cycle in both SA and RA walking (relative to LW) in the SMC. Additionally, we observed significant β rebound (synchronization) at the initial swing phase of the gait cycle, which may be indicative of active cortical signaling involved in maintaining the current locomotor state. An increase of low γ band power in this cluster was also found in SA walking. In the PPC, the low γ band power increased with the level of task demands (from LW to RA and SA). Additionally, our results provide evidence that electrocortical amplitude modulations (relative to average gait cycle) are correlated with the level of difficulty in locomotion tasks. Specifically, the modulations in the PPC shifted to higher frequency bands when the subjects walked in RA and SA conditions. Moreover, low γ modulations in the central sensorimotor area were observed in the LW walking and shifted to lower frequency bands in RA and SA walking. These findings extend our understanding of cortical dynamics of human walking at different level of locomotion task demands and reinforces the growing body of literature supporting a shared-control paradigm between spinal and cortical networks during locomotion.

## Introduction

The brain’s involvement in lower limb movements and gait has been studied extensively over the years using numerous imaging modalities [1–3], including functional magnetic resonance imaging (fMRI), functional near infrared spectroscopy (fNIRS), magnetoencephalography (MEG), and electroencephalography (EEG). Collectively, these studies have demonstrated a dynamic network of efferent (volitional) commands and afferent signaling (sensory feedback) for the modulation of bipedal locomotion, known as the central pattern generators (CPGs) [4, 5]. These networks were previously thought to be independent of the central nervous system (CNS), relying only on local signaling. However, research has shown that the spinal CPGs and locomotor regions of the brainstem are directly activated by cortical commands [6], including initiation and termination of gait from the supplementary motor area [7, 8] and in motor planning through visual feedback [9]. These studies support the theory of a shared-control paradigm for locomotor control, where cortical (supraspinal) networks dynamically modulate rhythmic motions originally initiated by spinal networks [10]. This growing body of literature has demonstrated that the cortex is directly involved in modulating locomotion through motor planning, execution, and error correction. However, understanding the cortex’s involvement in gait execution is challenging and requires the imaging method to be mobile. Thus, many studies using fMRI and traditional MEG (both stationary methods) have relied on gait imagery or observation [1–3] as a source for understanding cognition and gait. Many studies have shown that motor imagery and observation elicit responses from the same neuronal networks as actual task execution [11–17]; however, these studies are limited to stationary conditions and do not consider corticomuscular networks (networks connecting cortical brain activity and muscular activation) involved in the action. Furthermore, the studies utilizing fMRI are able to obtain a high spatial resolution, but sacrifice an important understanding of the evolving temporal dynamics of the associated neuronal networks. Thus, to understand the neural activity associated with task execution (i.e., information from the central and peripheral nervous systems), we must rely on mobile imaging modalities that provide temporal high resolution with respect to the action. Recent advancements in scalp electroencephalography (EEG) as a brain imaging modality have led to a greater understanding of the brain’s involvement in complex motor tasks. Specifically, the use of EEG has permitted the study of the neural dynamics involved in human locomotion during passive and active walking conditions with a very high temporal resolution [1–3, 18–27]. It is well understood that spectral dynamics in the cortex, specifically increases and decreases in spectral power, are related to movement onset and termination [28–31]. Power decreases, or event related desynchronization (ERD), in the μ-band (8–12 Hz) and β-band (14–30 Hz) are well-established correlates of movement imagination and execution [29], including the transition from sitting to standing [32] and from standing to walking [23]. Cortical involvement has been shown to play a significant role in passive walking (i.e., steady-state walking) [19–25], and in active walking, such as gait involving velocity changes [16–18, 33–36] and multi-tasking [37, 38]. Specifically, Gwin and colleagues observed significant spectral power modulations in the α and β bands in the anterior cingulate (AC), posterior parietal (PPC), and sensorimotor cortex (SMC) that were coupled directly to the gait-cycle (intra-stride modulations) [20]. Similar results have been shown in the SMC, PPC, and left and right motor areas during robot assisted walking and gait adaptation on a treadmill [24, 25]. Interestingly, the presence of θ (4–7 Hz) and γ (low: 25–40 Hz; high: 40–100 Hz) band oscillations have emerged as neural dynamics associated with challenging locomotor tasks, such as balance beam walking [27], walking in an interactive virtual environment [25], velocity control [18], and incline walking [26]. Increased θ band activity has been associated with error correction and appears to reflect the complexity of the task [18, 25, 26]. γ band oscillations have been associated with increased cortical computation [39, 40] and the coordination of functional cell assemblies [41], indicating that walking may require gait phase dependent neuronal synchronization for sensorimotor processing. All of these studies demonstrate the direct involvement of the cortex in walking, and further support the notion of shared-control between cortical and spinal networks during walking.

A deeper understanding of the neural dynamics of walking, especially on multiple terrains, has potential implications for use in brain-machine interfaces (BMIs) for control of rehabilitative and assistive robotic systems such as neuroprostheses. Previous studies have demonstrated the ability to detect human motor intent [23, 32, 42, 43], joint kinematics [44–48], and muscle activation patterns [49, 50] directly from time and/or frequency modulations of the cortex. However, an understanding of the neural correlates of over-ground multi-terrain walking is currently lacking. One of the limitations common to many of the previous studies [19–26] is the use of a treadmill, which has been shown to alter the biomechanics of locomotion when compared to over-ground walking [51]. Bulea and colleagues sought to address this using an active treadmill that adapts to the user’s walking speed, thus simulating over-ground walking conditions [18]. However, this paradigm does not allow for the investigation of cortical involvement in complex walking tasks, such as stair ascent and descent. In this study we seek to identify the neural dynamics of over-ground walking on level ground, ramps, and stairs using non-invasive mobile brain-body imaging (MoBI) EEG devices. We hypothesized that α and β band suppression will be greatest for stair walking followed by ramp walking. Furthermore, we expected significant increases in θ and γ band activity (especially for stair walking), reflecting heightened cortical processing (associated with increased physical work requirements and monitoring of errors) related to decreased task complexity (1. stairs, 2. ramps, 3. level walking).

## Materials and methods

### Experimental setup and procedure

**Fig 1:**
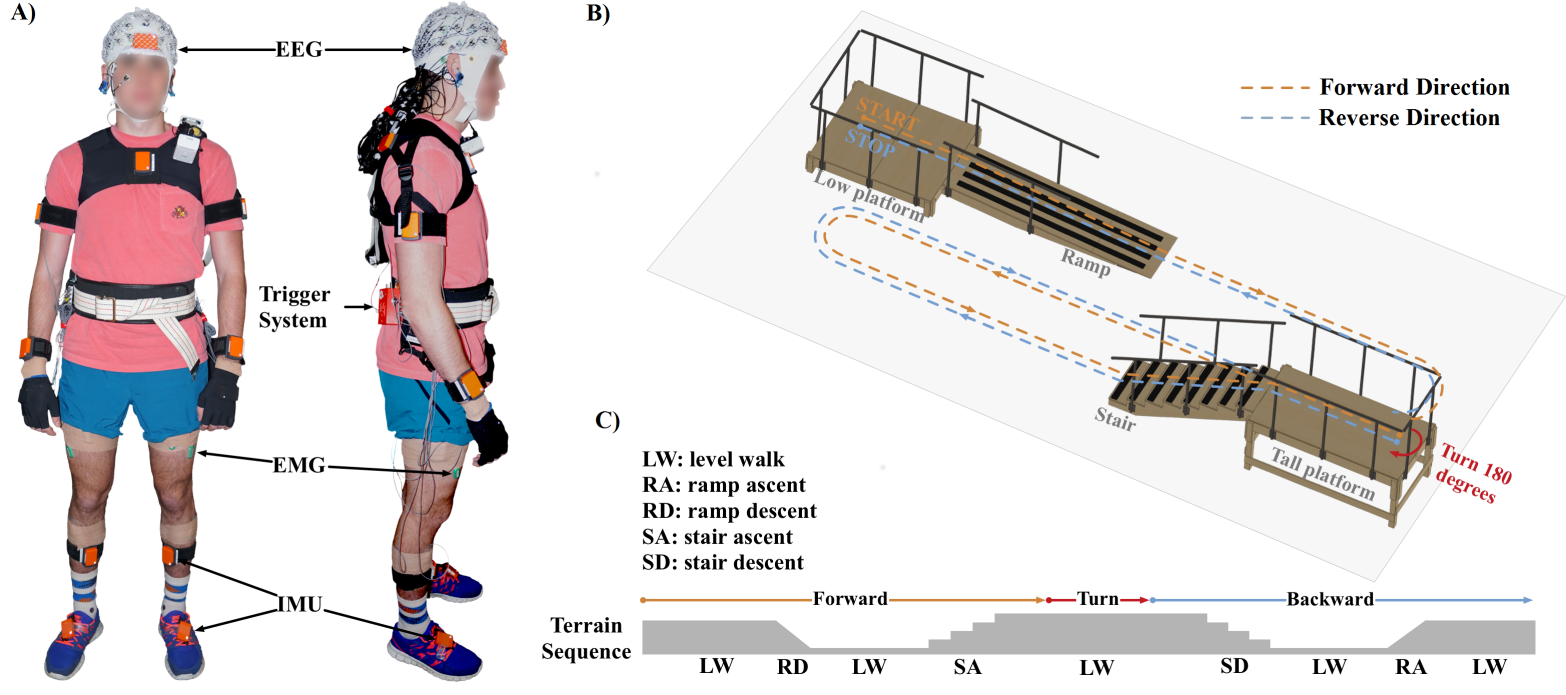
A) Experimental setup in this study. Each subject was instrumented with EEG, EMG, and IMU sensors and a trigger system. B) Illustration for gait course setup which was designed to provide five steady locomotion modes (level ground walking, stair descent, stair ascent, ramp descent, and ramp ascent). C) Experimental protocol. The subjects began walking, descended the ramp, transitioned to level ground walking, ascended the staircase (8 steps, step height: ~13.3 cm), and came to rest at the end of the stair platform (forward path). Ambulation back to the starting point (backward path) constituted one complete test trial.

Ten able-bodied healthy subjects (5 males; 5 females) enrolled in this study. All experimental protocols and informed consent (signed by all participants) were approved by the Institutional Review Board (IRB) at the University of Houston. All experiments were performed in accordance with the 45 Code of Federal Regulations (CFR) part 46 (“The Common Rule”), specifically addressing the protection of human study subjects as promulgated by the U.S. Department of Health and Human Services (DHHS). The individual in this manuscript has given written informed consent to publish these case details. Fig 1 shows the experimental setup and a diagram of the gait course used in this study. A 64-channel Ag/AgCl active electrode EEG setup (BrainAmp DC and MOVE, Brain Products GmbH, Germany) was used to record wirelessly at 1000 Hz from the face and scalp. Channels TP9, PO9, PO10, and TP10 were removed from the cap and used for electrooculography (EOG) to capture blinks and eye movements; however, these data were excluded from all analyses in this study. The remaining 60 channels were arranged according to the modified 10–20 international system, which was used in our previous study [50]. We used a 3-D electrode localization system (BrainVision Captrak, Brain Products GmbH, Germany) to record electrode positions. In addition to EEG and EOG, the subjects were instrumented with 17 wireless inertial measurement units (IMUs) for full-body motion capture (Xsens MVN, Xsens North America Inc., Culver City, CA). The IMU data was recorded at 30 Hz and time synchronized with the EEG using external hardware triggers. Muscle activities from the lower-limb were also acquired during the tasks. Surface EMG signals were recorded (Biometrics Ltd, Newport, UK) at 1000 Hz from twelve different sites (tibialis anterior, gastrocnemius, rectus femoris, vastus lateralis, bicep femoris long, semitendinosus from both legs). EEG, EMG and kinematics data collection were synchronized using a custom external hardware trigger system.

Five steady locomotion modes (level ground walking, stair descent, stair ascent, ramp descent, and ramp ascent) were designed into the experimental course. Subjects were instructed to walk at their preferred walking speed. The subjects began level walking, descended the ramp, transitioned to level ground walking, ascended the staircase, and came to rest at the end of the stair platform (forward direction; red path in Fig 1). The subject would then turn around 180°, descend the stairs, transition to level ground, ascend the ramp, and come to rest (reverse direction; blue path in Fig 1). Ambulation in both the forward and reverse direction of the course constituted one complete test trial (Fig 1). The subjects completed an average of 20 trials.

### EEG Signal processing and source localization

**Fig 2:**
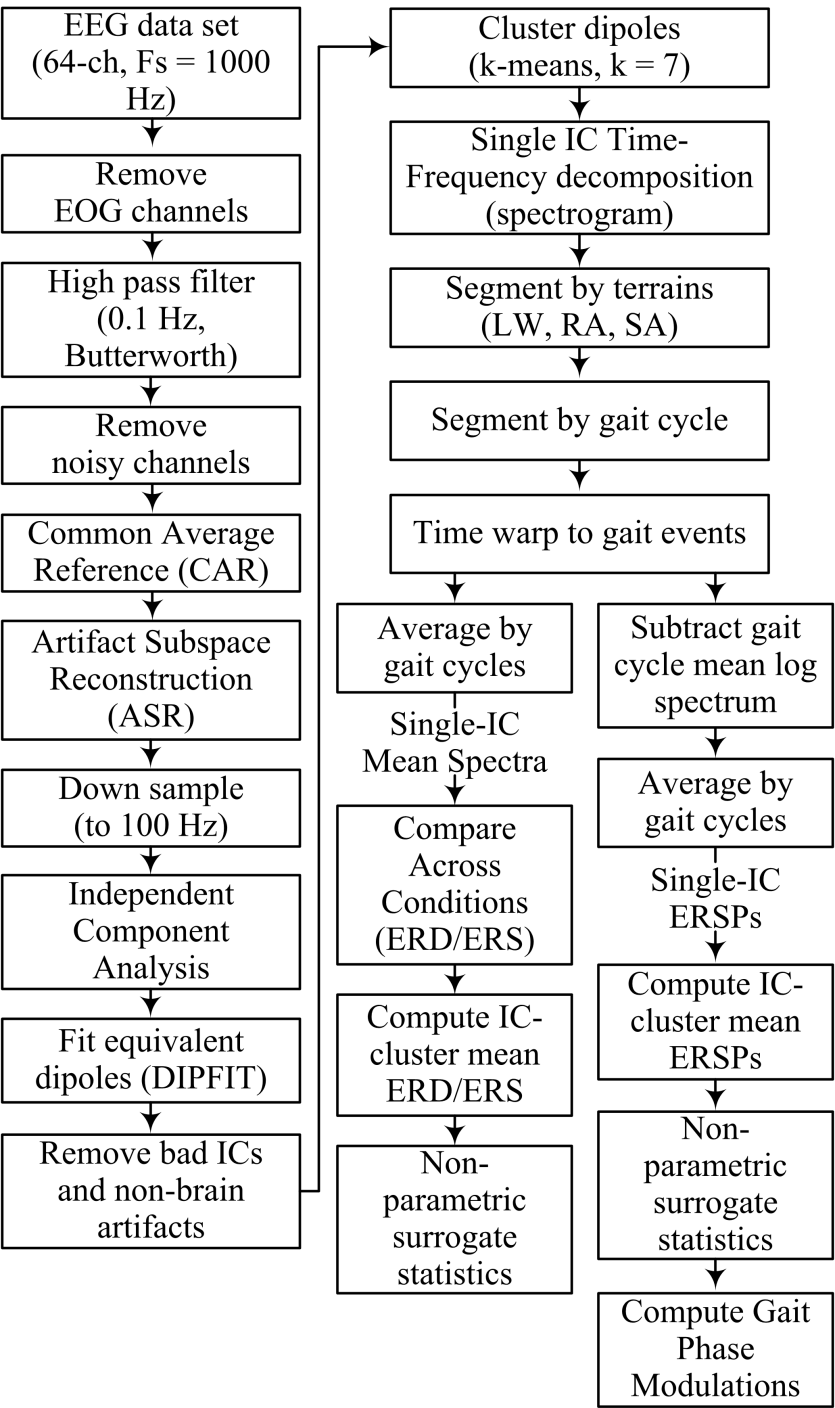
Flow-chart indicating EEG data-processing pipeline.

EEG signal processing and statistical analysis were performed using custom software written in Matlab R2016a (The MathWorks, MA) and functions from EEGLAB [52]. A flowchart outlining the EEG signal processing pipeline is shown in Fig 2. EOG channels were first removed and the remaining EEG signals (60 channels) were high pass filtered at 0.1 Hz using a 4^th^ order Butterworth filter. Corrupted EEG channels, indicated by having a standard deviation greater than 1000 μV or kurtosis of more than five standard deviations from the mean, were rejected [20]. The remaining EEG channels were then re-referenced by subtracting to their common average. Next, artifact subspace reconstruction (ASR) was applied to remove high amplitude artifacts (e.g., eye blinks, muscle burst) [18]. In this study, one min of EEG recorded during quite standing at the beginning of each session was used as baseline (calibration) data for ASR. A sliding window (length of 500 ms) and a variance of three standard deviations were used to identify corrupted subspaces. After this step, EEG data were down-sampled to 100 Hz and Infomax independent component analysis was applied.

EEG electrodes were aligned to a standard MNI brain model (Montreal Neurological Institute, Quebec, Canada) by using 3D position data obtained from a Captrak system. We then computed equivalent current dipole that matched to the scalp projection of each independent component (IC) source by using a standard three-shell boundary element head model included in the DIPFIT toolbox [52]. Only ICs in which the equivalent dipoles explained > 80% of variance of the IC scalp projections were retained for further analysis. Next, we visually inspected each IC scalp projection, its equivalent dipole’s location, and its power spectra and removed ICs that related to non-brain artifacts (e.g., eye blink/movement, neck muscle). We generated feature vectors (which include power spectra, IC scalp projections, and dipole locations) from the remaining ICs and used the k-means algorithm (k = 7) to obtain IC clusters across subjects. ICs that were further than three standard deviations from a cluster centroid were categorized into an outlier cluster and omitted from further analysis. Details for rejection of bad channels and identification of non-brain ICs in this study are provided in the supplementary materials.

Time-frequency decomposition (spectrogram) was performed using short-time Fourier transform for each IC in the clusters (window length of 500 ms, maximum overlap, and the time index at the beginning of the segment window) across the whole trial. The full-length spectrogram was then segmented using the kinematic data into different walking conditions (LW, RA, SA) and gait cycles. An average gait event template for each condition was obtained by averaging the time between adjacent gait events across all subjects and all trials. The specific events were right heel contact to left to off, left toe off to left heel contact, left heel contact to right toe off, and right toe off to right heel contact. The spectrogram epochs were time-locked to the average gait events across all trials so that each gait event occurs at the same latency. A single spectrogram was obtained for each IC (single-IC) by averaging the time-locked spectrograms for each gait cycle within each walking condition. To compare spectrograms across walking conditions, we normalized by subtracting the average time frequency spectrum during level walking (baseline) from the average spectra for the ramp ascent and stair ascent conditions. Next, a single spectrogram was obtained for each condition from each of the cortical clusters by averaging the normalized spectrograms across all ICs. The resulting spectrogram for each condition, cluster-IC, resulted in a single spectrum showing ERD/ERS compared to the level-ground walking condition. Significant ERD/ERS values were masked for significance (*α* = 0.05) using a non-parametric bootstrapping technique with random shuffling of 200 surrogate data [52].

To compute intra-stride power modulations within each IC (known as *event related spectral perturbations*—ERSP), the log mean of each spectrum (across time, resulting in a 1x*frequency* column vector) was subtracted from the log spectrogram at each time point. The mean normalized log spectrograms were then stacked into a 3D matrix and averaged across gait cycles to obtain single-IC ERSPs. Finally, we obtained IC-cluster mean ERSPs for each waking conditions by averaging single-IC ERSPs across all ICs. Significant ERSPs (*α* = 0.05) were identified using a bootstrapping technique within EEGLAB [52].

We utilized the gait phase modulation (GPM) measure to identify the EEG frequency components with amplitude modulations most strongly locked to the gait cycle. The GPM measure has recently been proposed to analyze the modulation of EEG oscillatory amplitudes relative to the gait cycle [53]. In this study, we computed GPM values based on the definition suggested by Trenado [54].

$${GPM}_{f}=\frac{2}{N\sqrt{2}\sigma_{A}(f)}\left|\sum_{n=0}^{N-1}{A(n,f)e}^{-2\pi i\frac{2n}{N}}\right|$$ (1)

where *N* is the number of samples per gait cycle, *A(n*,*f)* denotes time-frequency magnitude at given frequency *f* and sample *n*, and *σ*_*A*_(*f*) denotes its standard deviation.

## Results

**Fig 3:**
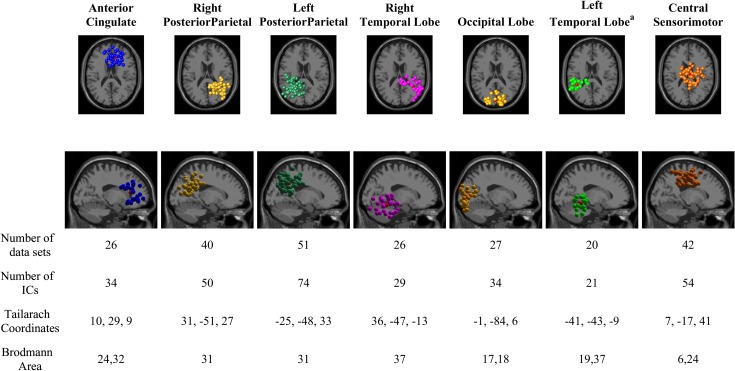
Clusters of dipolar sources fit to independent components for all subjects across all trials, which includes all walking conditions (LW, RA, and SA). Brodmann areas are the regions found within ±5mm search range of cluster centroids. ^a^ These clusters did not cover a majority of the subjects, and where excluded from further analysis.

The total number of gait cycles retained for analysis from each condition was 1724, 846, and 626 for LW, SA, and RA, respectively. The k-means clustering resulted in six cortical areas: the right and left posterior parietal cortex (PPC), sensorimotor cortex (SMC), anterior cingulate (ACC), the right and left temporal lobe (TL), and the occipital lobe. The clusters from the left temporal lobe did not cover a majority of the subjects and thus were omitted from further analysis. Fig 3 shows the number of data sets and the number of IC sources contained in each cluster. Information regarding the cluster centroid, such as the Talairach coordinates and Brodmann areas, which were identified from the Talairach atlas [55], are also illustrated in Fig 3. The Brodmann areas were searched within ±5 mm cube ranges around each cluster centroid. The electrocortical activity in the PPC and central SMC clusters were further analyzed to compare the changes of time-frequency spectrograms and amplitude modulations (relative to the mean gait cycle) across different walking conditions. The PPC and central SMC areas are known to be directly involved in interlimb coordination during locomotion [56–58].

**Fig 4:**
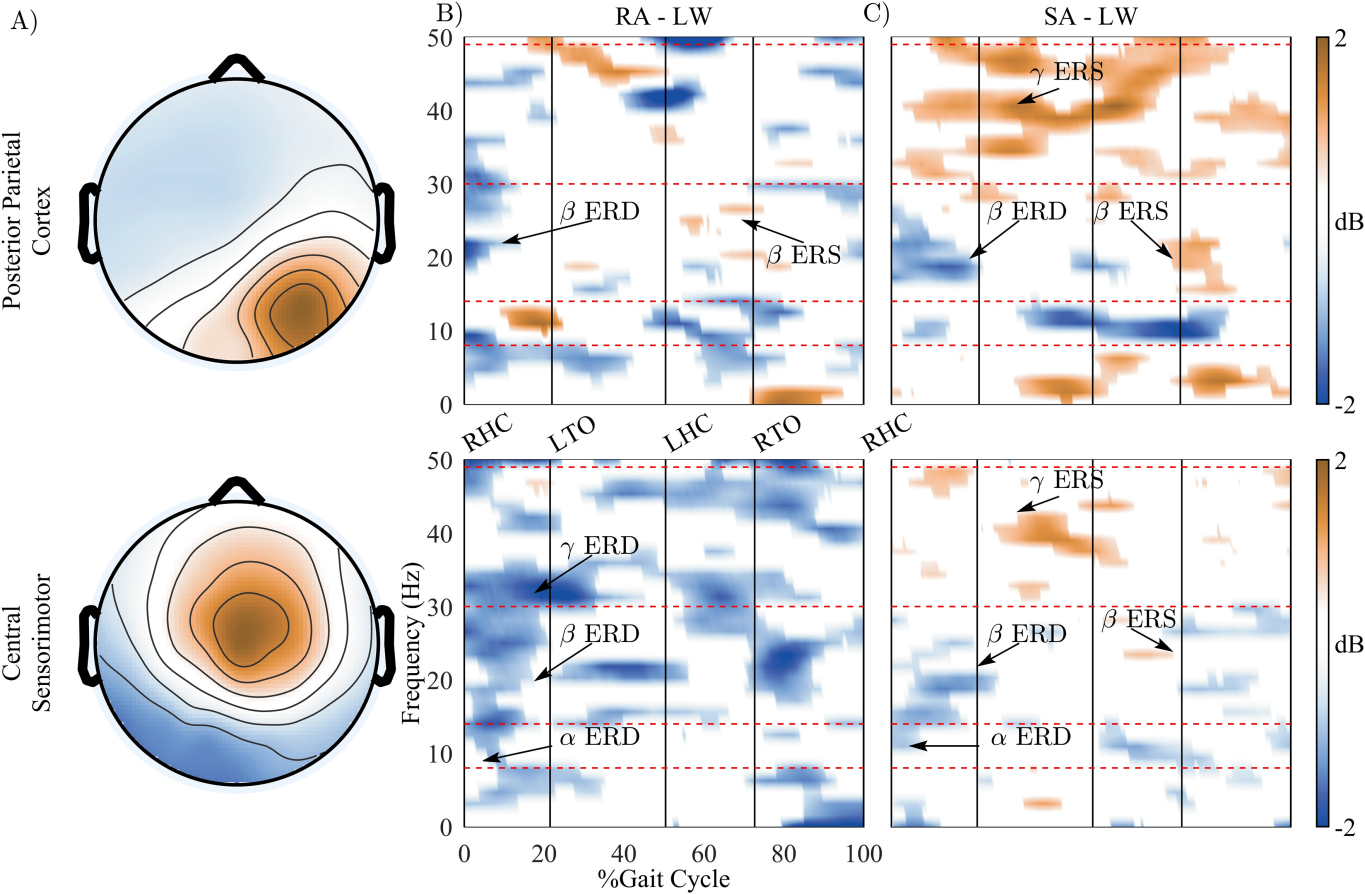
The changes of time-frequency spectrogram across different walking conditions. A) Topographical scalp projections of ICs comprising in the Posterior Parietal Cortex and the Central Sensorimotor clusters. B) and C) show power changes (relative to level-ground walking periods) in the ramp ascent (RA) and the stair ascent (SA) walking conditions, respectively. Displayed are only significant power changes (p < 0.05). The blue color indicates significant power decrease (ERD) and the brown color indicates power increase (ERS).

We observed significant differences of time-frequency spectrograms when the subjects walked in different walking conditions (LW, RA, and SA). Fig 4B and 4C show power changes (relative to level-ground walking periods) of the PPC and central sensorimotor (SMC) clusters in the RA and the SA walking conditions, respectively. Displayed are only significant power changes (p < 0.05). Significant power decreases (ERD) in the α (8–13 Hz) and β (14–30 Hz) band in the SMC were observed at the beginning of gait cycle (from RHC to LTO) for both the RA and SA conditions. Similar decreases of β power in the PPC were found in both conditions, with the β ERD being more sustained during SA. The β ERD in RA and SA returned to normal activation levels directly following the toe off event in both the PPC and SMC. Interestingly, Fig 4 reveals increases of β power (ERS or β rebound) at the end of the gait cycle (after RTO) for both RA and SA in the PPC and for SA in the SMC. The β rebound occurred after significant decreases of β power (PPC cluster in RA and SA condition, and SA in SMC cluster). The β rebound in the SMC occurred directly prior to the right toe off event, during the initial swing period.

A substantial increase of power (ERS) in the low γ (30–49 Hz) band was observed in the PPC for the SA condition, which was sustained across a large section of the gait cycle. Low γ ERS in the SMC was also found in SA condition. However, such increased power in the low γ band was not found in the SMC or PPC in RA condition.

**Fig 5:**
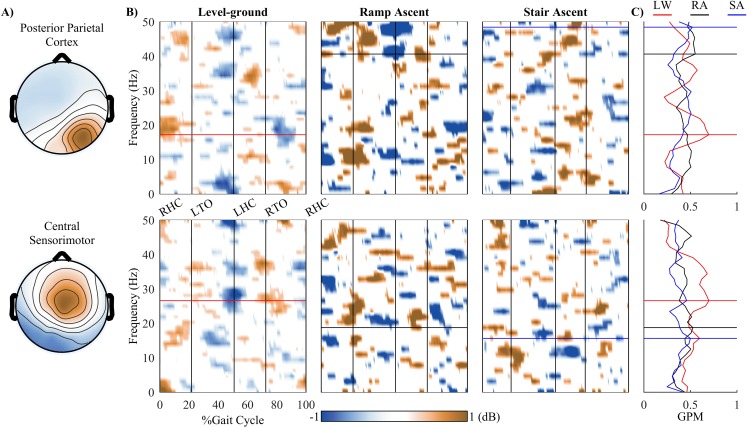
Event related perturbations (ERSPs) and gait phase modulations (GPM) for each cluster in different walking conditions (LW, RA, and SA). A) Topographical scalp projections of ICs comprising in the PPC and central sensorimotor clusters. B) Grand average ERSPs for different walking conditions. ERSPs plot were masked with significant values using a bootstrapping technique within EEGLAB (*α* = 0.05). C) GPM values for different walking conditions.

Fig 5B shows the presence of amplitude modulations (relative to the mean gait cycle) in the low γ band in the PPC during RA and SA walking. This low γ band modulation was not found in the LW walking condition. Additionally, gait phase modulation (GPM) peak frequency was found at ~18 Hz (β band) in LW and the peak frequency shifted to higher values in the low γ band during RA (~42 Hz) and SA (~48 Hz) walking conditions. On the other hand, the modulations in the SMC were most pronounced in the low γ band during LW walking. When walking in RA and SA conditions, the modulations in this cluster decreased in the low γ and increased in the β bands. The GPM peak frequency in this cluster shifted from low γ band (~28 Hz) to the β band in RA (~18 Hz) and SA (~16 Hz) walking conditions.

## Discussion

Whereas previous studies have analyzed cortical activity during typical treadmill walking, walking with a robotic assisted gait trainer, or with varying gait speeds using an active treadmill, this study is the first to examine electrocortical dynamics of human walking across different unconstrained walking conditions (i.e., level ground, ramp ascent, and stair ascent). Source localization using blind source separation (i.e., ICA) and k-means clustering revealed the involvement of seven clusters during the walking tasks. Our results showed that the changes of spectral power in the PPC and SMC cluster are associated with the level of motor task demands. Additionally, our results presented evidence that electrocortical amplitude modulations (relative to average gait cycle) are correlated with the level of difficulty in locomotion tasks. These findings extend our understanding of cortical dynamics of human walking at different level of locomotion task demands.

Our results showed decreased power in the α and β band (ERD) in the RA walking (relative to LW) in the central sensorimotor cortex (Fig 4). Significantly decreased power in the α and β bands have been shown in recent studies when participants were actively involved in the walking tasks [18, 24]. Bulea et al. suggested that the decreases in power in the α/μ and β bands indicate increased cortical involvement when participants walked on an active treadmill or with increased gait speed. Therefore, our results further support previous findings, suggesting that α and β ERD in the SMC are greater and more sustained at the beginning of gait cycle as the physical demands of human locomotion increase. Additionally, we observed β ERD in the PPC during RA and SA. The β ERD occurred at the beginning of gait cycle and it was more pronounced and sustained for SA than in RA, possibly indicating greater activation of underlying cortical networks related to the physical requirements and/or complexity of the task. The presence of significant β ERD in the PPC was also reported in a previous study which compared active and passive walking to quite standing [18].

A particularly interesting finding in our study is the presence of β ERS, or rebound, around the right toe off event in the gait cycle. A decrease in β band activity is a correlate of movement preparation and execution, which is replaced by increased activation of faster rhythms, such as γ, during continuous or complex movement [59]. This is evident in our data (PPC) during the loading-response phase of the gait cycle during SA, where β activity is suppressed while γ band activity is simultaneously increased. A previous review of β band oscillations has proposed that increases/decreases in neural β activity may reflect the maintenance or interruption of currently active motor processes [59]. In other words, decreases in β band activity may reflect voluntary action, while increases in β activity may be a signal to maintain the current state. Thus, in both RA and SA, β ERD in the loading response may be related to active propulsion through the gait cycle, while β rebound, or increased β activity, may relate to processing the upcoming heel strike leading into the future gait cycle.

Our results demonstrated increased γ synchronization during SA in both the PPC and SMC. As previously mentioned, we observed β ERD at the beginning of the gait cycle for both clusters and both conditions. During the SA conditions, we observe gait-phase dependent γ ERS during loading response, and following the left toe off, left heel contact, and right toe off events. This same pattern of γ band ERS was observed in the SMC, but with a smaller increase in power compared to the PPC and primarily leading up to the left heel contact event. γ band activity has been associated with increased cortical computation [39, 40] and may reflect increased cortical involvement in the motor task. Furthermore, the PPC and SMC areas have been associated with visuomotor processing and are directly involved in interlimb coordination during locomotion [56–58]. In our results, the precise alignment of γ ERS to gait events may be an indication of cortical computation related to limb coordination, increased proprioceptive feedback, and visuomotor motor processing. Prior studies have shown γ ERS during double-support, which may indicate proprioceptive feedback that is used to prepare for the next single-support phase [27]. The results of ERD/ERS in the β and γ bands indicate significant sensorimotor processing during the RA and SA conditions, where the cortical activations reflect movement onset and muscle loading, while also providing efferent signaling for both proprioceptive feedback and as a switch to maintain the current motor state—i.e., continue ramp/stair locomotion. Furthermore, these results may indicate that these conditions require increased cortical involvement that is proportional to the complexity of the walking task.

It is well established that cortical spectral fluctuations are aligned to gait cycle events [1–3, 18–27]. Indeed, our results reinforce these findings: specifically, β, γ, and θ were modulated with the timing of gait cycle events. We observed that the frequency of the cortical oscillations shifts to different bands based on the locomotor task. Seeber et al. [21] introduced the notion of gait phase modulations (GPM)—later modified by [54]—as a metric for quantifying the frequency components with amplitudes most strongly associated with the gait cycle. They identified low γ (24–40 Hz) modulations in the central sensorimotor to be significantly aligned to gait cycle events. In our study, we observed the GPM peak frequency in the SMC to be around 28 Hz, agreeing with these previous findings. γ band activity has been reported to increase during phasic movements involving isotonic muscle contractions [21, 60]. Bulea et al [18] hypothesized that shifts in peak frequency to the γ band enables the integration of visual and somatosensory feedback during complex walking tasks. During LW, we observed shift towards γ; however, during RA and SA, we observed a shift towards β band GPM in the central sensorimotor.

The PPC has been clearly linked to voluntary movements through the processing of somatosensory, proprioceptive, and afferents. Activity in the PPC has been demonstrated to reflect interlimb coordination during locomotion and has been proposed to represent kinesthetic feedback related to muscle activity [26]. Indeed, we observed a shift in peak modulation frequency to γ during RA and SA compared to LW. Increased muscle activations and differences in kinematics during RA and SA are likely attributed to greater activity in the PPC. Furthermore, the peak frequency in the PPC shifted to ~42 Hz during RA and ~48 Hz during SA; the increase between RA and SA is further evidence of increased involvement of the PPC in locomotion with increased biomechanical demands. Bulea et al. posited that gait-phase specific γ activations in PPC may demonstrate increased attention to foot velocity [18], based on the association of γ band synchronization with increased cortical computation [39, 40].

Though significant findings of EEG correlates of human walking have been predominantly studied in the delta Δ (0.1–3 Hz) [49, 61–63], α/μ (8–13 Hz) [18, 24], β (14–30 Hz) [53], and low γ (30–49 Hz) [20, 24, 53], a limitation of this study is the bandwidth used for analysis which was constrained to 50 Hz and below. Extending the analysis to the high-gamma range (e.g., 50–100 Hz) would be desirable in future studies to study more localized gait-related modulations of EEG. Our results showed neural correlates of human walking across different unconstrained walking conditions corresponding to different level of motor task demands. Future work is needed to determine if these features could be used for assessing the level of voluntary control of human gait by monitoring electrocortical activities from non-invasive EEG signals. Voluntary control of movements is crucial for motor learning and physical rehabilitation [64, 65]. Therefore, such assessment could be beneficial for neurological gait rehabilitation. The findings in this study also have implications for developing a noninvasive brain-computer-interface system for intuitive and flexible control of lower-limb neuroprostheses under different locomotion modes. Future studies will consider designing such interface for the next generation of advanced lower-limb neuroprosthetic devices.

## Supporting information

S1 File Supplementary information for the removal of bad EEG channels and the rejection of non-brain independent components during the process of EEG cleaning. (PDF)
