# Supplementary material for: Electrocortical correlates of human level-ground, slope, and stair walking
Source: PLoS One. 2017 Nov 30;12(11):e0188500. doi: 10.1371/journal.pone.0188500 (PMC5708801; doi:10.1371/journal.pone.0188500)
Supplement: S1 File — (PDF) [file pone.0188500.s001.pdf]

Supplementary Material

Electrocortical Correlates of Human Level-ground, Slope, and Stair walking

Trieu Phat Luu, Justin A. Brantley, Sho Nakagome, Fangshi Zhu, and Jose L. Contreras-Vidal

*** Correspondence:** Trieu Phat Luu: ptluu2@central.uh.edu

In this supplementary material, we represent the rejected channels and independent components (ICs) during the process of EEG cleaning.

Supplementary information for channel removal

EEG data for six subjects, nine trials for each, were processed. On average, two channels per trial were identified as bad channels and were rejected during the noisy channel removal process. The maximum number of channels removed in a single trial was seven channels, and the minimum was zero.

Supplementary figures explaining rejected independent components using residual variance rejection

In this section, we report the statistics and the characteristics of the rejected ICs with a supplementary figure. **S1 Fig.** shows an example of rejected ICs as an outcome of cleaning after applying DIPFIT function. The figure was obtained by using EEGLAB toolbox function "pop_selectcomps" (Delorme and Makeig, 2004). The number on top of each topoplot represents the index of ICs and the percentage in brackets next to the index shows the residual variance (RV) value. All the ICs with RV of more than 20% were rejected and on average 39 ICs (67%) of the ICs were removed in this process.

- Rejected poor fit ICs (RV > 20%)
  - An average number of ICs rejected per subject per trial: 38 ICs (67% of total ICs)
  - Maximum number of ICs rejected per subject per trial: 50 ICs (87% of total ICs)
  - Minimum number of ICs rejected per subject per trial: 21 ICs (36% of total ICs)

Supplementary figures explaining rejected non-brain independent components

This section explains the rejected non-brain ICs with their characteristics shown in the supplementary figures

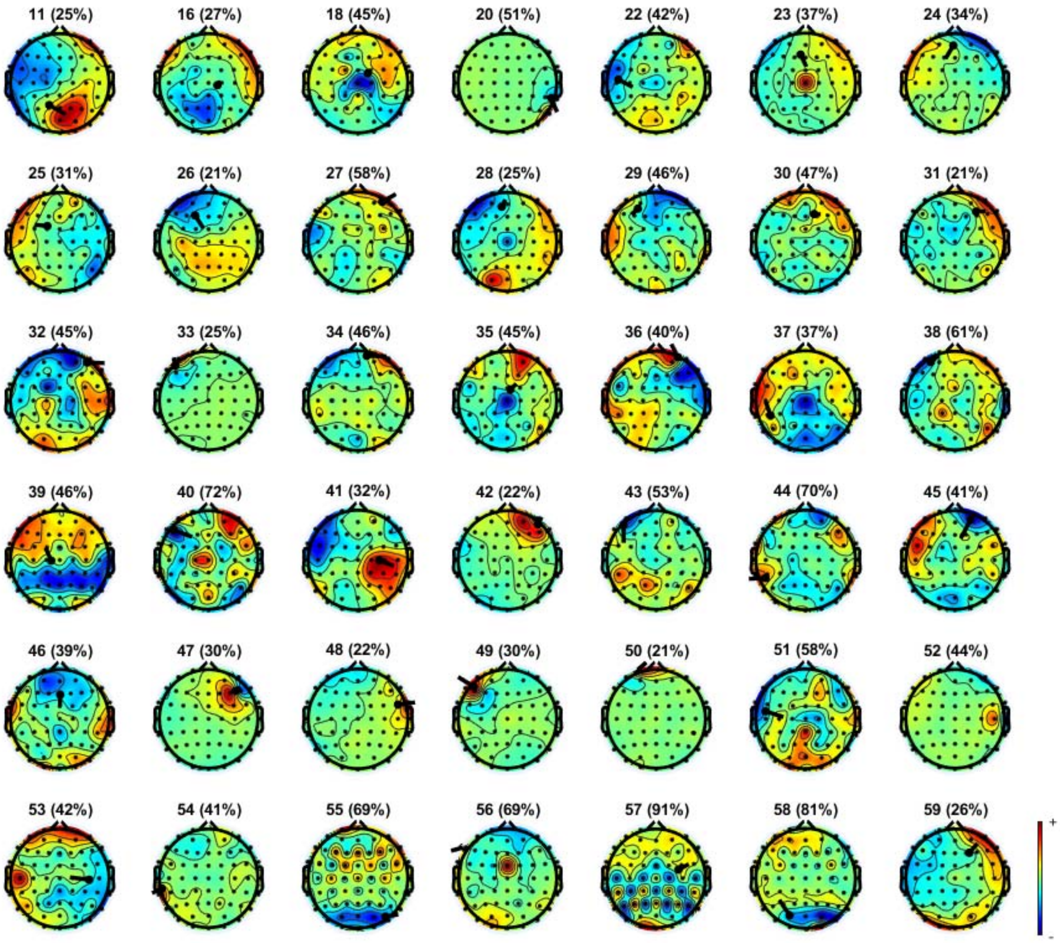

S1 Fig. An example of rejected ICs with residual variance of more than 20%. The number on the top of each topoplot shows the index of ICs, followed by the residual variance in brackets.

S2 to S4 Figs. show examples of manually removed nonbrain-ICs after the removal of ICs in the first process. The MRI image on the left side shows the identified dipole location as well as the strength and the direction of the dipole. The topoplots on the right side shows the 2D topoplot and the power spectrum. We used dipole locations identified on the MRI image on top of given features in the Graphical User Interface (GUI) provided by the EEGLAB to carefully select the artefactual components.

The S2 Fig represents eye blinks component where the frontal part of the scalp is strongly affected and the rest of the topoplot shows no change in the weights. It typically shows a smooth decreasing power in the activity power spectrum. In addition, the dipole is located at the eye in MRI image in the sagittal plane. The S3 Fig shows eye movement component where the frontal part of the scalp is symmetrically activated between left and right. Again, the dipole is also located at the eye in MRI image in the sagittal plane. The removal of eye related artifact ICs were represented in more detail in the previous studies (Jung et al., 2000a;Jung et al., 2000b). At last, S4 Fig shows muscle artifact IC where we could see a high power in the higher frequencies. In addition, it is known to be spatially

localized around the peripherals. Furthermore, the dipole location shown in the MRI image from axial plane shows the dipole is located at the outer side of the cortex. A detailed explanation for each artefactual ICs were also represented in this paper (Chaumon et al., 2015).

- Rejected non-brain ICs
    - An average number of ICs rejected per subject per trial: 15 ICs (26% of total ICs)
    - Maximum number of ICs rejected per subject per trial: 33 ICs (57% of total ICs)
    - Minimum number of ICs rejected per subject per trial: 6 ICs (10% of total ICs)

Therefore, remaining ICs are calculated by (A total number of ICs) – (ICs rejected during RV > 20%) – (ICs rejected as non-brain artifacts).

- Remaining ICs
    - An average number of ICs remained per subject per trial: 4 ICs (7% of total ICs)
    - Maximum number of ICs rejected per subject per trial: 8 ICs (14% of total ICs)
    - Minimum number of ICs rejected per subject per trial: 1 ICs (2% of total ICs)

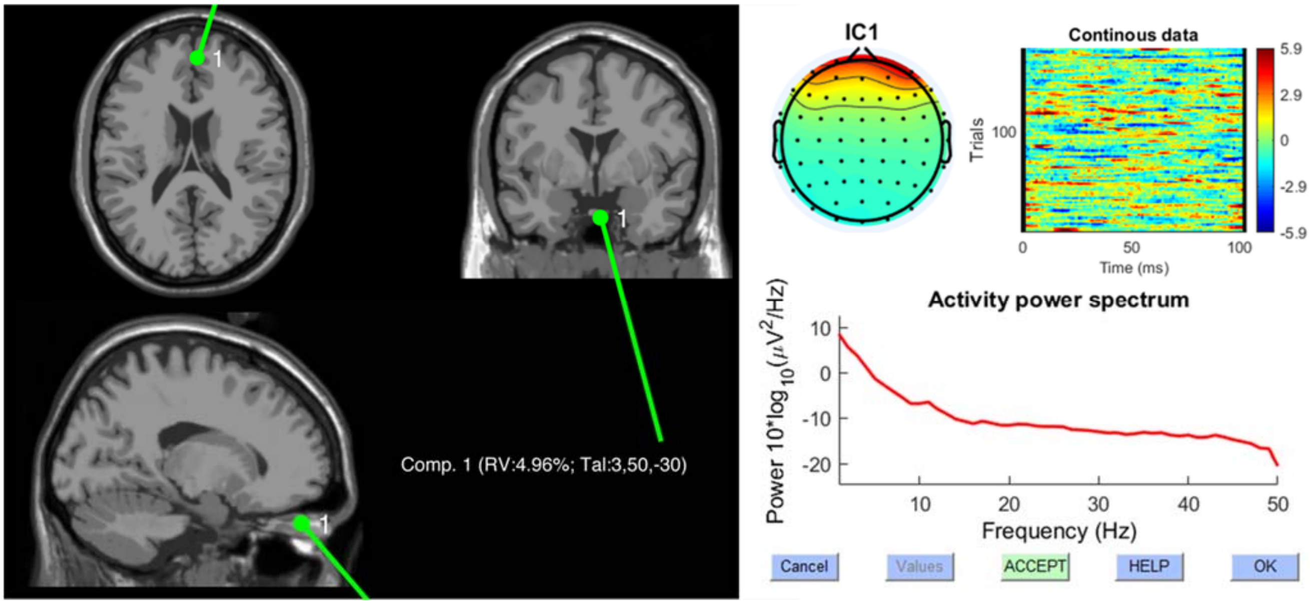

S2 Fig. An example of rejected non-brain IC for eye blink. The left MRI plots show the dipole location and the strength with directions. The right plots show the topoplot of the IC with the activity power spectrum.

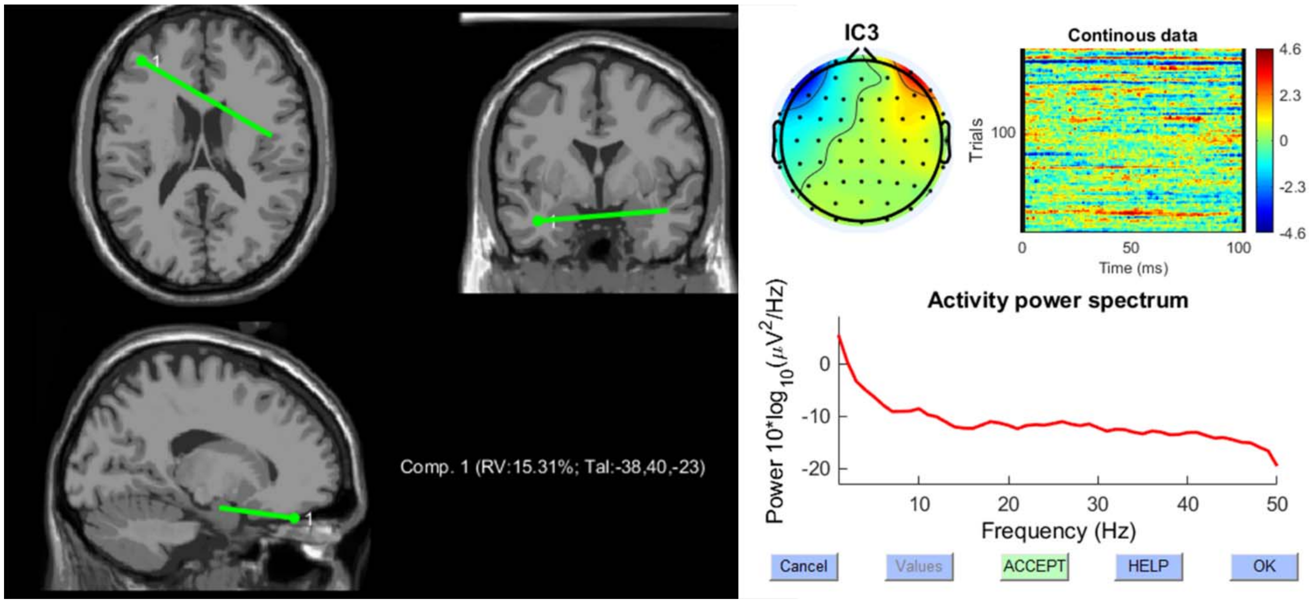

S3 Fig. An example of rejected non-brain IC for eye movement.

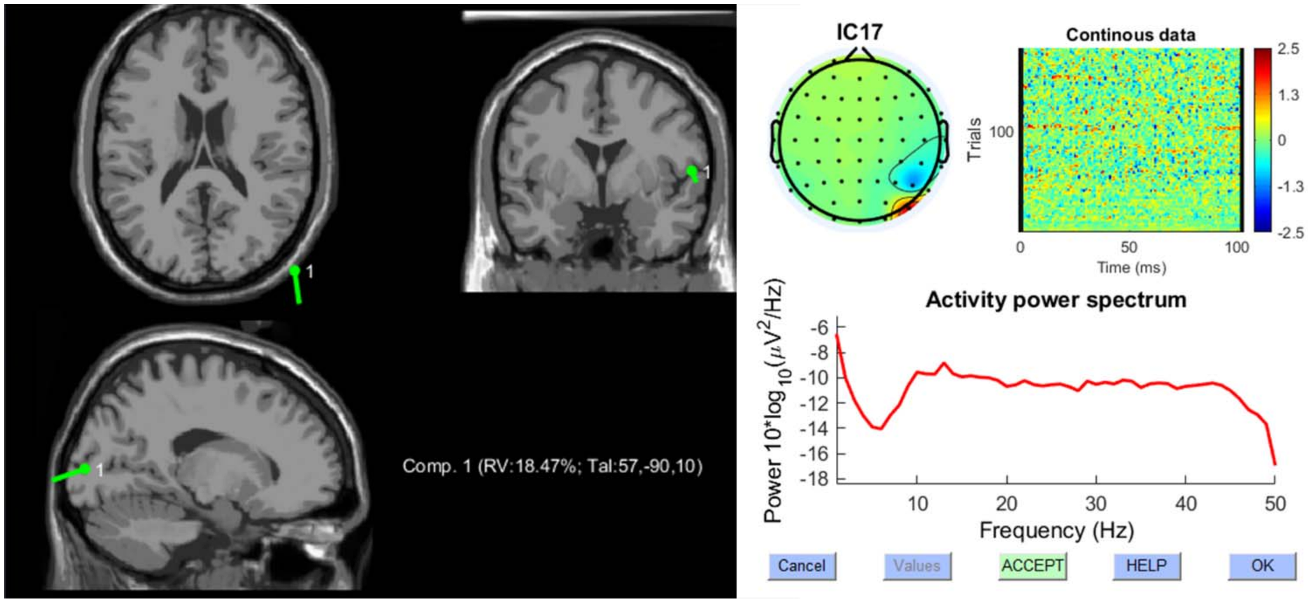

S4 Fig. An example of rejected non-brain IC for EMG.

References:

Chaumon, M., Bishop, D.V., and Busch, N.A. (2015). A practical guide to the selection of independent components of the electroencephalogram for artifact correction. *Journal of neuroscience methods* 250, 47-63.

Delorme, A., and Makeig, S. (2004). EEGLAB: an open source toolbox for analysis of single-trial EEG dynamics including independent component analysis. *Journal of neuroscience methods* 134, 9-21.

Jung, T.-P., Makeig, S., Westerfield, M., Townsend, J., Courchesne, E., and Sejnowski, T.J. (2000a). Removal of eye activity artifacts from visual event-related potentials in normal and clinical subjects. *Clinical Neurophysiology* 111, 1745-1758.

Jung, T.P., Makeig, S., Humphries, C., Lee, T.W., Mckeown, M.J., Iragui, V., and Sejnowski, T.J. (2000b). Removing electroencephalographic artifacts by blind source separation. *Psychophysiology* 37, 163-178.
